# Seed physiological traits and environmental factors influence seedling establishment of vegetable soybean (*Glycine max* L.)

**DOI:** 10.3389/fpls.2024.1344895

**Published:** 2024-06-03

**Authors:** Xiaoying Li, Kathryn Liu, Steven Rideout, Luciana Rosso, Bo Zhang, Gregory E. Welbaum

**Affiliations:** ^1^ School of Plant and Environmental Sciences, Virginia Tech, Blacksburg, VA, United States; ^2^ Department of Horticultural Sciences, Tropical Research and Education Center, University of Florida, Homestead, FL, United States

**Keywords:** vegetable soybean (edamame), genotypes, seed leachate, temperature, seedling emergence, LabField™

## Abstract

Edamame (*Glycine max* (L.) Merr.), a specialty soybean prized for its nutritional value and taste, has witnessed a surge in demand within the U.S. However, subpar seedling stands have hindered its production potential, necessitating increased inputs for farmers. This study aims to uncover potential physiological factors contributing to low seedling emergence in edamame. We conducted comprehensive assessments on thirteen prominent edamame genotypes alongside two food-grade and two grain-type soybean genotypes, focusing on germination and emergence speed in both laboratory and field settings. Additionally, we employed single electrical conductivity tests and identified and quantified seed leachate components to distinguish among soybean types. Furthermore, using a LabField™ simulation table, we examined seed emergence across a wide soil temperature range (5°C to 45°C) for edamame and other soybean types. All seeds were produced under the same environmental conditions, harvested in Fall 2020, and stored under uniform conditions to minimize quality variations. Our findings revealed minimal divergence in emergence percentages among the seventeen genotypes, with over 95% germination and emergence in laboratory conditions and over 70% emergence in the field. Nonetheless, edamame genotypes typically exhibited slower germination speeds and higher leachate exudates containing higher soluble sugars and amino acids. Seed size did not significantly impact total emergence but was negatively correlated with germination and emergence speed, although this effect could be mitigated under complex field conditions. Furthermore, this study proposed differences that distinguish edamame from other soybean types regarding ideal and base temperatures, as well as thermal time. The finds offer valuable insights into edamame establishment, potentially paving the way for supporting local edamame production in the U.S.

## 1 Introduction

Vegetable soybean, also known as “edamame,” is a specialty soybean [*Glycine max* (L.) Merr.] that is harvested at the R6 growth stage (i.e., reproductive stage when the pods are 85%-90% filled with green seeds) (Ogles et al., 2016). Unlike grain-type soybean, which is mainly grown for oil production and animal feed, edamame is consumed directly by humans. Although edamame has been consumed in East Asian countries for centuries, its global popularity has surged in recent decades, notably in the United States. Currently, edamame ranks as the second most popular soy product in the U.S., trailing only behind soymilk (Hartman et al., 2016). Sales have soared from 18 million USD in 2003 to 30 million USD in 2007, reaching 84 million USD by 2013 (Hartman et al., 2016), with a consistent annual growth in consumption of 12–15% (Neill and Morgan, 2021). This escalating demand has motivated growers to contemplate domestic production of this specialty vegetable.

Despite the U.S. being a leading producer of grain-type soybean, the journey towards establishing a thriving domestic edamame production industry in the United States has encountered significant hurdles, with poor seedling emergence being a paramount concern. Williams (2015) reported an average emergence below 35% among 136 diverse edamame cultivars representing most commercial and public cultivars available in the U.S., which is much lower than a normal plant population (80%) for commercial grain soybeans (Sánchez et al., 2005). The seedling establishment issue has also been observed in edamame field trials in many states in the U.S., including North Dakota, Georgia, Illinois, Pennsylvania, and Virginia, where emergence percentages range from 60-85% for different cultivars (Sánchez et al., 2005; Williams and Bradley, 2017; Sutton et al., 2020). In some instances, the yield loss can be partially offset by the increased branching of plants when an intermediate rate of emergence occurs. However, significant yield loss would occur if a large proportion of seeds fail to emerge, especially under adverse environmental conditions.

To achieve a successfully stand of edamame, it is crucial to use locally adapted cultivars with high-quality seeds. In response to this necessity, breeders have dedicated their efforts to developing new edamame cultivars tailored for U.S. production systems. Laboratory tests play a pivotal role in this process, as they are employed to evaluate the quality of these cultivar seeds and are usually used to predict their future performance in the field. Most of the tests, such as Association of Official Seed Analysts (Association of Official Seed Analysts, 2018), are conducted in controlled laboratory settings optimized for species-specific germination, assessing a seed’s ability to germinate. Additionally, rapid germination, characterized by the swift emergence of radicles, serves as a sensitive indicator of seed vigor, aiding in mitigating risks associated with biotic and abiotic stresses in the soil (Marcos Filho, 2015). Other critical tests, such as electrical conductivity (EC), which measures ion leakage during seed imbibition and is directly linked to the disruption of cell membrane systems and permeability control (Powell, 1986), has been also widely adopted to evaluate seed vigor in various crops for decades (Vieira et al., 2004).

Edamame is bred for its large bean size and high sugar content, contributing to the appealing appearance and taste, which is favored by consumers in the U.S. (Li et al., 2022). However, it is unknown whether these desirable traits exhibit a negative inverse relationship with germination and emergence. Moreover, little is currently known about growing edamame in the U.S. Edamame growers currently rely heavily on production recommendations for grain-type soybeans, despite the larger seed size and higher sugar content of edamame. Many assumptions made about growing edamame stem from a lack of data (Williams et al., 2022). Thus, the objectives of this study are: 1) to evaluate the germination and seedling establishment of the edamame genotypes newly developed by the breeding programs of Virginia Tech and the University of Arkansas; 2) to identify the underlying physiological traits contributing to variations in establishment ability between edamame and other soybean types (i.e., food-grade and grain-type soybeans); and 3) to determine the differences in emergence response to temperature between edamame and other types of soybeans. Our research endeavors to enhance understanding of the critical factors influencing edamame stand establishment, providing a foundation for proper crop management decisions, and ultimately contributing to the advancement of edamame production in the United States.

## 2 Materials and methods

### 2.1 Plant materials

Thirteen newly developed edamame breeding lines from programs at Virginia Tech (referred to as V lines) and the University of Arkansas (referred to as R lines) were compared. Additionally, we included two released edamame cultivars, namely “VT Sweet” and “UA-Kirksey,” as reference varieties, commonly known as “checks” in plant breeding studies (Chen et al., 2014; Zhang et al., 2022). These genotypes were carefully chosen based on their desirable edamame traits, including large seed size, appealing pod appearance, and high sugar and protein content. We also incorporated two food-grade natto-type cultivars, MFS 48P1 and MFS 561, along with two grain-type soybean cultivars, Ellis and USG 5618V, as commercial reference checks. Detailed information on all 17 genotypes used in this study is provided in **Supplementary Table S1**. Seed size was determined by calculating the mean weight of 100 randomly selected whole seeds from each genotype across three replications (**Supplementary Table S1**). All seeds were produced in Blacksburg, VA, during the fall of 2020, harvested, conditioned, dried, and stored under uniform conditions to minimize variations in seed quality affecting germination performance. All seed samples were cleaned by hand, removing moldy, mottled, discolored, or off-types seeds.

### 2.2 Seed viability and vigor evaluation

#### 2.2.1 Germination test

The viability of various seed genotypes was assessed through a modified germination test based on the AOSA criteria (Association of Official Seed Analysts, 2018), employing a complete randomized design (CRD) consisting of 25 seeds per replication and a total of four replications for each genotype (Galarza, 2000; Zhou et al., 2016). Seeds were uniformly spaced on two layers of germination blotter paper (Hoffman Manufacturing Co. in Albany, Oregon), saturated with 18 mL of deionized water in the acrylic germination boxes (11.2 × 11.2 × 3.5 cm high; feet on the bottom of the boxes were removed by filing). Boxes were sealed and placed on a one-dimensional thermogradient table (Thermogradient Systems, Blacksburg, VA) maintained at 25 ± 1°C in the dark. Boxes were monitored and randomized daily within each temperature. Germination was scored as radicle emergence when exceeding the seed length. Germinated seeds were carefully removed and counted using tweezers. Throughout the testing, deionized water was added to the germination boxes as necessary to maintain optimal moisture levels. Both the final germination percentage and germination speed were evaluated.

#### 2.2.2 Seedling emergence test

The soil emergence experiment of 17 different edamame and other soybean genotypes was conducted using the LabField™ (Thermogradient Systems, Blacksburg, VA) simulation table. The table evaluates seedling emergence, more accurately by simulating field conditions compared to germination on blotting paper (Welbaum et al., 2016). Rows on the table were filled with Promix potting soil (Miracle-Gro, Lawn Products Inc., USA) and the soil temperature was adjusted to 25 ± 1°C. Edamame seeds were planted 3 cm depth (Zhang et al., 2013). After planting, the soil surface was lightly compressed using a wooden block and irrigated with deionized water to maintain soil moisture. The experimental design was a completely randomized within each temperature with 15 seeds per replication and two replications for each genotype and temperature. The entire experiment was replicated twice. Seed counts were conducted every 24 hours for 10 days. Seeds were considered to have emerged when the coleoptile had fully emerged from the soil, and normal seedlings were removed and counted accordingly. Both final emergence percentage and emergence speed were evaluated.

#### 2.2.3 Field test

In May 2021, a field experiment was conducted at the Virginia Tech, Kentland Research Farm in Whitethorne, Virginia, to assess the field emergence of 17 genotypes. Each genotype was planted in four replications, and the plots were arranged in a randomized complete block design. A total of 50 seeds were planted in a single-rows 2.74 m long spaced 0.8 m apart. A planting depth of approximately 3 cm was used for both grain-type and vegetable soybeans (Zhang et al., 2013). The experiment relied solely on rainfed conditions, with no irrigation applied during the entire growth season. Emergence counts were conducted at the V2 to V3 growth stage, characterized by the presence of one to two unrolled trifoliate leaves of individual plants. Seedling counts were made every 24 hours for a duration of four weeks, continuing until no further emergence of new seedlings was observed. Both final emergence percentage and emergence speed were evaluated. Temperature sensors were placed at seed depth in the middle of each row to record soil temperature hourly throughout the experiment.

### 2.3 Seed sugar quantification of the seventeen genotypes

Sucrose, raffinose, and stachyose are the predominant sugars found in soybean seeds. A prior study by Vandecasteele et al. (2011) suggested a potential negative correlation between seed vigor traits and the sucrose/raffinose ratio in the legume plant *Medicago truncatula*. Sucrose, raffinose, and stachyose were extracted from 17 genotypes to assess possible effects on germination (Lord et al., 2021). In brief, seeds were initially ground to a fine powder using a water-cooled grinder. Each sample was accurately weighed 0.1 g, after which 1.0 mL of high-performance liquid chromatography (HPLC)-grade water was added to each sample. The samples were then shaken vigorously for 15 minutes at 400 strokes per minute. Following this, the samples were centrifuged at 13.2 rpm for 15 minutes, and 0.5 mL of the supernatant was transferred to new 2.0 mL centrifuge tubes. Subsequently, 0.7 mL of acetonitrile (ACN) was added, and all tubes were thoroughly mixed by inverting them multiple times. The samples were allowed to sit at room temperature for 1 hour and were then centrifuged at 17,000× g for 15 minutes. After centrifugation, 100 μL of the supernatant was mixed with 900 μL of 65% ACN and filtered through a 0.2 μm membrane into an HPLC sample vial. The concentrations of sucrose, stachyose, and raffinose were determined using HPLC in accordance with Lord et al., 2021. Three samples were tested for each genotype, and each sample underwent two technical replicates.

### 2.4 Seed leachate evaluation

#### 2.4.1 Conductivity test

Electrical conductivity (EC) was measured on individual seeds to mitigate undue influence of leakage from mechanically damaged seeds on bulk seed measurements. In this method, individual seed along with 6.0 mL of deionized water was placed into a 17 mm diameter tube (Corning Inc., USA) (Mattioni et al., 2015). Tubes were incubated in a temperature control cabinet at 25 ± 1°C for 24-hour incubation period. Seeds with visibly damage were replaced prior to conductivity measurements. After incubation, a meter (FiveGo™ F3, Mettler Toledo, USA) measured electrical conductivity at 24h of each tube in μS·cm^−1^g^−1^ seed (Kulik and Yaklich, 1982). All of the seventeen genotypes were tested, and each genotype was replicated 25 times.

Considering the marked differences in electrical conductivity observed between edamame and other type soybeans during the seed conductivity test conducted above, six representative genotypes selected from different soybean types were used for a comprehensive assessment of changes in conductivity values over time. These genotypes encompassed three edamame genotypes, including one breeding lines (V16-0521) and two commercial checks (UA Kirksey and VT Sweet), along with two commercial food-grade cultivar (MFS 48P1 and MFS 561), and one grain-type soybean cultivar (USG 5618V). The leachate samples were prepared using the same method as in the EC test. Ten to twenty replicates from each genotype were subjected to a 36-hour soaking period (until conductivity values reached a stable phase) at 25 ± 1°C. Deionized water was used as the negative control. The electrical conductivity of the imbibition solution was manually recorded at three-hour intervals throughout the process.

#### 2.4.2 Identification and quantification of seed leachate components

In this study, ten different genotypes were analyzed, including five edamame genotypes (V16-0688HP, V16-0521, V16-0523HP, R17-2965, and VT Sweet), as well as two commercial food-grade cultivar (MFS 48P1 and MFS 561), along with two grain-type soybean cultivars (Ellis and USG 5618v). This selection was made based on their diverse EC values and to ensure representation of each soybean type. The leachate samples were collected using supernatant from the EC test protocol (i.e., samples were collected after 24 hours of seed soaking). Three leachate samples were assessed for each genotype, and each of these samples underwent two technical replicates, with deionized water serving as the negative control.

While EC provided insights into ion leakage from seeds, it lacked specificity concerning the identify and quantities of ions present. Qualitative and quantitative analysis of minerals Ca, P, Na, K, Mg in the leachate were determined by inductively coupled plasma atomic emission spectrometry (ICP-AES) at the Virginia Tech Soil Testing Laboratory in Blacksburg, Virginia. Furthermore, the analysis of sugars and amino acids in the leachate was conducted using HPLC-ELSD (evaporative light scattering detection) based on the methodologies outlined by Shanmugavelan et al. (2013) and Singer et al. (2022), respectively. The positive sugar controls included stachyose, sucrose, raffinose, maltose, glucose, mannose, xylose, fructose, galactose, and arabinose, while positive amino acid controls encompassed aspartic acid, glutamic acid, serine, histidine, glycine, threonine, arginine, cysteine, tyrosine, alanine, leucine, lysine, isoleucine, phenylalanine, tryptophan, methionine, and valine.

### 2.5 Emergence response to temperature

Thermogradient tables can assess germination responses to various temperatures (Schwember and Bradford, 2005; Zhou et al., 2016; Wang et al., 2020). However, germination experiments on moistened paper in containers on a thermogradient table are not always good predictors of field emergence because edaphic factors from soil are lacking. LabField™ tables are similar in concept to thermogradient tables but utilize soil with gussets welded perpendicular to the table surface to control temperatures (Welbaum et al., 2016). To provide a more realistic assessment of genetic differences that may affect field emergence of edamame, LabField™ tables were used to compare germination performance at different temperatures.

To establish and maintain a continuous temperature gradient in soil along the table surface, refrigerated and heated baths (Brookfield AMETEK, Inc., Middleboro, MA, USA) circulated warm and cool ethylene glycol solutions through plumbing welded to the bottom and on opposite ends of each LabField™ simulation table. The area between gussets on the table surfaces was filled with Promix potting soil (Miracle-Gro, Lawn Products Inc., USA). Temperature uniformity across the width of the table, varying by less than 1°C, allowed for the placement of replications at specific temperatures across the table.

Two LabField tables were utilized to establish a total of 17 different soil temperatures ranging from 5°C to 45°C. One table provided experimental temperatures at 5°C, 7°C, 10°C, 13°C, 16°C, 19°C, 21°C, and 23°C, while the other provided temperatures of 25°C, 27°C, 29°C, 32°C, 35°C, 38°C, 41°C, and 45°C. Soil temperatures were monitored according to Zhou et al. (2016). Button-style miniature temperature loggers (Watch Dog, Temp 2K, B-Series; Spectrum Technologies, Inc., Aurora, IL, USA) were placed among the seeds in the soil. These loggers were positioned in at least two locations within each row for accurate hourly temperature data collection. Edamame cultivars VT Sweet and UA-Kirksey, both widely cultivated in the Mid-Atlantic region, were chosen as representative genotypes, along with two other types of soybeans, i.e., one food-grade (MFS 48P1) and one grain-type (USG 5618v) cultivar, as controls for testing on LabField™ tables. VT Sweet and UA-Kirksey were selected due to minimal variation among the other 13 genotypes in previous germination, emergence, and field tests. The experimental design employed a completely randomized layout with 15 seeds per replication and four replications per cultivar at each temperature. Seed counts were recorded every 24 hours until no emergence was observed for three consecutive days. Seeds were considered to have fully emerged when the hypocotyl had emerged entirely from the soil. Emerging seedlings were carefully removed from the soil using forceps. Soil moisture levels were consistently maintained at optimal conditions through regular visual and tactile assessments. Both final emergence percentage and emergence speed were evaluated.

### 2.6 Statistical analysis

Performance measures were derived from seed germination and emergence, focusing on two aspects: the speed and percentage of germination and emergence, following the methodology outlined in Zhou et al. (2016): Germination/Emergence Percentage (GP/EP) = (S/total seeds planted) × 100; while Mean Germination/Emergence Time (MGT/MET) was determined as: MGT/MET = ∑T_i_N_i_/S and expressed as days per seed. Here, T_i_ represents the number of days after sowing, N_i_ represents the number of seeds that germinated or emerged on day i, and S represents the total number of germinated seeds or emerged seedlings.

To identify possible genetic differences among genotypes and to assess the impact of temperature on seedling emergence, mean base temperatures (T_b_) for emergence were determined by extrapolating the linear portion of mean ER (Emergence Rate) versus temperature (T) plots to the abscissa intercept. ER was determined as the reciprocal of MET and expressed as seeds per day (d^−1^). The slopes of regression lines were used to calculate the reciprocal of thermal times to emergence (1/θ_T_), allowing for the comparison of emergence speed across different temperatures. The mean maximum temperature (T_m_) was estimated as the temperature that reduced emergence percentage to 50% since the ER versus T plot was nonlinear at higher temperatures. Optimal temperatures (T_o_) for emergence were identified where the highest emergence percentage and ER coincided (Zhou et al., 2016).

Measurements for all tests in this research were carried out with a minimum of three replicates. ANOVA analysis was again performed in the R statistical package (version 4.0.2, https://www.r-project.org/) using the Tukey method to compare genotype differences at p<0.05. Pearson’s correlation coefficient (r) and probability (p) values were determined using the t-test in the R. To ensure homogeneity of variance, an arcsine transformation was applied to the percentage data, and a log transformation was applied to the time values prior to analysis (Jett et al., 1996). Untransformed values were shown in tables and figures.

## 3 Results

### 3.1 Germination and emergence of edamame

#### 3.1.1 Germination and seedling emergence tests

The final germination percentages exceeded 80%, at the optimal temperature 25°C (Association of Official Seed Analysts, 2018) for the thirteen edamame genotypes while V10-3653 and R17-2776, reaching 100% (**Table 1**). The MTG, of these edamame genotypes ranged from 4.4 to 5.6 days. Notably, there were no significant differences observed for both germination percentage and MTG among these edamame genotypes. When comparing these edamame genotypes with food-grade soybeans and grain-type soybeans, no differences were found in the final germination percentages among the different soybean types. However, edamame generally exhibited significantly lower MTG compared to other types, which ranged from 2.6 to 3.5 days to germinate. There were no significant differences observed between food-grade and grain-type soybeans in terms of both germination percentage and MTG.

**Table 1:** Germination and emergence of the newly developed edamame genotypes and other soybean types.

Genotype Name	Germination Test^L^		Seedling Emergence Test^L^		Field Establishment	
	MGT (days)	AGP (%)	MET (days)	AEP (%)	MFET (days)	AFEP (%)
*Edamame*						
V16-0518	4.8a	95.0ab	3.6abc	99.0a	10.0a	81.5a
V16-0688HP	4.8a	93.0ab	3.4abc	99.0a	9.1a	86.0a
V16-0527	4.8a	99.0ab	3.4abc	100.0a	10.0a	88.5a
V16-0521	5.6a	91.0ab	3.3abc	100.0a	9.1a	89.5a
V16-0523HP	5.3a	88.0ab	3.4abc	98.0a	10.0a	80.0a
V17-0621ED	5.3a	84.0b	4.0a	97.0a	10.0a	86.0a
V10-3653	4.6ab	100.0a	3.4abc	100.0a	10.0a	79.5a
R15-10280	4.8a	97.0ab	3.7ab	95.0a	10.0a	85.5a
R17-2750	4.8a	99.0ab	3.8ab	99.0a	9.1a	89.0a
R17-2776	4.4abc	100.0a	3.8abc	99.0a	9.1a	82.5a
R17-2965	5.3a	96.0ab	3.6abc	98.0a	10.0a	79.0a
VT Sweet	4.8ab	98.0ab	3.6abc	95.0a	10.0a	82.5a
UA Kirksey	4.4abc	98.0ab	3.6abc	100.0a	10.0a	83.5a
*Food grade soybean*						
MFS 48P1	2.6d	100.0a	3.0c	100.0a	10.0a	73.0a
MFS 561	3.5bcd	91.0ab	3.3bc	94.0a	9.1a	82.0a
*Grain type soybean*						
Ellis	3.2d	100.0a	3.7abc	93.0a	10.0a	75.0a
USG 5618v	3.3cd	99.0ab	3.1c	98.0a	9.1a	80.5a

Values are means of at least three experiments with 15-100 seeds per genotype per experiment. Means followed by the same letters are not significantly different according to Tukey’s HSD (P <0.05) within the data collected in the same column. MGT: Mean Germination Time; AGP: Average Germination Percentage; MET: Mean Emergence Time; AEP: Average Emergence Percentage; MFET: Mean Field Emergence Time; AFEP: Average Field Emergence Percentage; L: experiments conducted under laboratory condition.

All seventeen genotypes, regardless of soybean type, displayed satisfactory seedling emergence (>90%) when planted at the optimal soil temperature of 25°C, with no differences in emergence percentage observed among them. Moreover, there were no noticeable disparities in the MTG between edamame genotypes or between edamame and other soybean types. All MTG ranged from 3.1 to 4.0 days (**Table 1**).

#### 3.1.2 Field test

Both edamame and grain-type soybeans emerged on the 8^th^ day after planting, with no further emergence observed beyond the 24^th^ day after sowing. Throughout these 24 days, the soil temperature ranged from 10-21°C at night and 22-40°C during the daytime, with average daily temperatures ranging from 15-26°C (see **Supplementary Figure 1**). The total precipitation in these days was 4.9 cm (Weather Underground, 2024).

There were no significant differences in field emergence percentage or MTG among the edamame genotypes or grain soybean. The percentages ranged from 73% to 89%, and the MTE for all genotypes averaged around 10 days, for both edamame and grain soybeans. All edamame genotypes exhibited a high MTE, consistently reaching or exceeding 80%, suggesting successful establishment of edamame genotypes is possible (**Table 1**).

### 3.2 Seed sugar quantification of the seventeen genotypes

To explore the relationship between seed performance and sugar content, we analyzed the three major sugars, i.e., stachyose, sucrose, and raffinose from seeds of the seventeen genotypes. Sucrose emerged as the predominant sugar, featuring an average content of 4.9% (dry weight basis), followed by stachyose at 3.1%, with raffinose registering the lowest at 0.6%. Notably, all three sugars displayed variations among the edamame genotypes. Specifically, for stachyose, V16-0688HP exhibited the highest content at 3.7%, while V16-0527 had the lowest at 2.5%. Raffinose content peaked in V16-0523 at 0.9%, followed by R17-2750 at 0.8%, and V10-3653 with the lowest at 0.5%. Regarding sucrose, V16-0523HP had the highest sucrose content at 6.4%, while V10-3653 had the lowest at 4.0%. Moreover, except for sucrose, there were no discernible distinctions among different soybean types in terms of these three sugars or the ratio of raffinose to sucrose. Edamame consistently exhibited higher sucrose levels, ranging from 4.0% to 6.4%, compared to other soybean types, which ranged from 2.9% to 4.4% (**Supplementary Table S2**).

### 3.3 Seed electrical conductivity

#### 3.3.1 Seed conductivity tests

**Figure 1:**
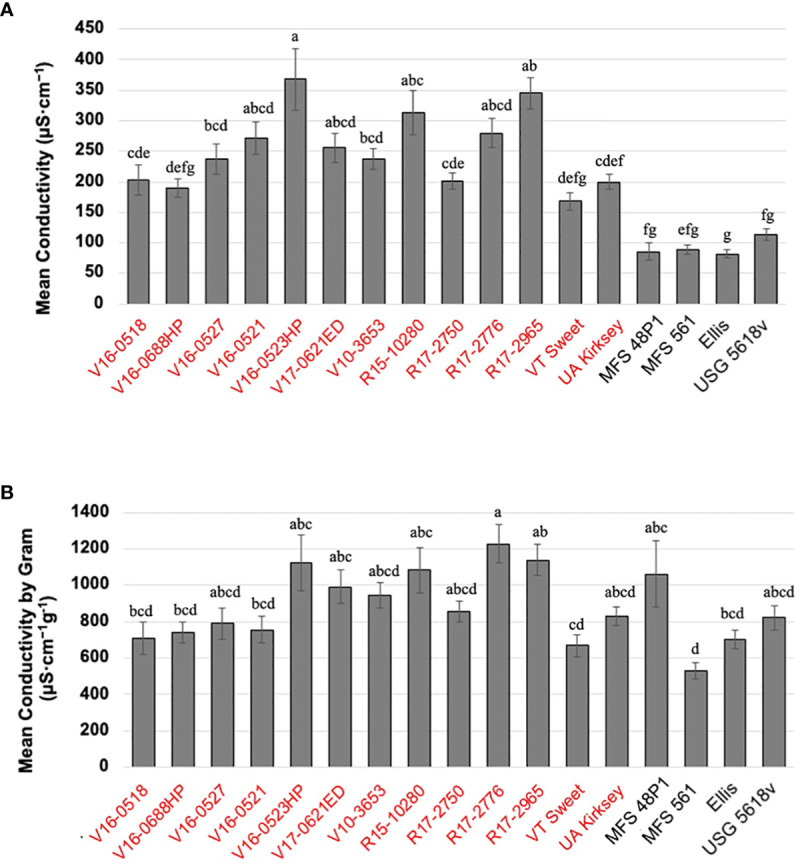
Mean individual electrical conductivity of the seventeen genotypes **(A)** and mean individual electrical conductivity by seed weight **(B)**. The figures display means and standard error deviations (SE), which were calculated from 25 replicative measurements for each genotype. The same letters are not significantly different according to Tukey’s HSD (P <0.05). Genotypes highlighted in red belong to the edamame, while the remaining genotypes belong to other soybean types.

Edamame genotypes exhibited a range of leachate conductivity values, spanning from 168.2 μS·cm^-1^ to 367.8 μS·cm^-1^. V16-0523HP displayed the highest conductivity, while VT Sweet demonstrated the lowest, followed by V16-0688HP at 190. 1 μS·cm^-1^, and the commercial edamame cultivar UA Kirksey at 199.6 μS·cm^-1^ (**Figure 1A**). In comparison, both food-grade and grain-type soybeans displayed consistently lower conductivity levels, ranging from 82.0 to 114.3 μS·cm^-1^, with no noticeable differences observed among genotypes within these categories.

Given the significant variations in EC values detected between edamame and other types of soybeans due to their seed size variations, a thorough evaluation of the changes EC values over a 36-hour soaking period was conducted using three representative edamame and three other soybean genotypes. The three edamame genotypes exhibited a similar pattern in their conductivity time-courses (**Supplementary Figure S2**). Within the initial 9 hours, conductivity increased rapidly, and all three genotypes reached over 70% of their final values as they entered the stationary phase. Subsequently, conductivity continued to rise, albeit at a slower rate, until it stabilized at 24 hours. Notably, at the 36-hour mark following seed soaking, the breeding line V16-0521 recorded a conductivity value of 229.5 μS·cm^-1^, while the two commercial edamame cultivars, VT Sweet and UA Kirksey, displayed values of 168.9 μS·cm^-1^ and 200.7 μS·cm^-1^, respectively.

In contrast, both food-grade and grain-type soybeans exhibited similar trends. Their conductivity increased rapidly during the initial 0-3 hours, reaching over 50% of their final conductivity values by 36 hours. Subsequently, conductivity increased gradually, and by 9 hours of soaking, it had reached over 70% of the final values as they entered the stationary phase, stabilizing at 24 hours which is same to the edamame genotypes. At the 36-hour, the food-grade MFS 561 and the grain-type soybean USG5681v displayed similar values, measuring 98.8 μS·cm^-1^ and 100 μS·cm^-1^, respectively. However, another food-grade soybean, MFS 48P1, exhibited a lower value of 51.8 μS·cm^-1^, possibly due to its smaller seed size compared to the others.

Given the substantial variation in seed size among genotypes, which could contribute to differences in electrical conductivity (EC), we normalized the individual seed conductivity by seed weight (g) to compare EC values per seed weight across all genotypes. Consequently, the disparities between edamame and other soybean types diminished, and EC values varied by genotype, ranging from 528.7 μS·cm^-1^ g^-1^ to 1,226.0 µS·cm^-1^ g^-1^. The food grade soybean MFS 561 exhibited the lowest conductivity per seed weight, followed by the commercial edamame cultivar VT Sweet at 667.3 µS·cm^-1^ g^-1^, while R17-2776 displayed the highest value (**Figure 1B**).

### 3.4 Identification and quantification of seed leachate components

#### 3.4.1 Soluble sugars

**Figure 2:**
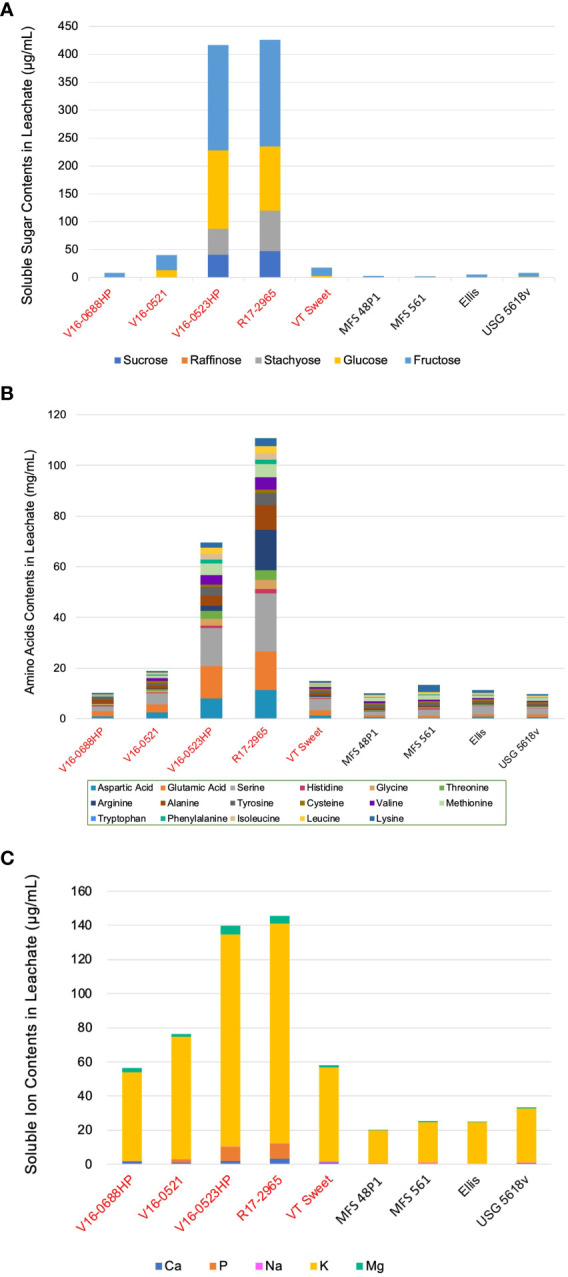
Identification and quantification of soluble sugars **(A)**, amino acids **(B)**, and mineral elements **(C)** in the seed leachate of nine representative genotypes. Genotypes highlighted in red belong to the edamame, while the remaining genotypes belong to other soybean types.

Five edamame genotypes (V16-0688HP, V16-052, V16-0523HP, R17-2965, and VT Sweet), two food-grade soybeans (MFS 48P1 and MFS 561), and two grain-type soybeans (Ellis and USG 5618v), spanning the range of conductivity among the seventeen genotypes, were selected to identify and quantify the sugar composition of seed leachate collected after 24 hours. In the soybean leachate, four soluble sugars, including sucrose, stachyose, glucose, and fructose, were identified (**Figure 2A**). While raffinose was not detected.

All genotypes contained fructose, with concentrations ranging from 8.4 μg·mL^-1^ to 191.1 μg·mL^-1^ for edamame, 2.6 μg·mL^-1^ to 3.2 μg·mL^-1^ for food-grade soybeans, and 5.1 μg·mL^-1^ to 6.9 μg·mL^-1^ for grain-type soybeans. Sucrose and stachyose were only found in the leachate of R17-2965 and V16-0523HP at concentrations of 40.9 μg·mL^-1^ and 47.7 μg·mL^-1^ for sucrose, and 46.2 μg·mL^-1^ and 72.5 μg·mL^-1^ for stachyose, respectively. Glucose was present in four out of the five edamame genotypes, with concentrations ranging from 3.6 μg·mL^-1^ to 140.5 μg·mL^-1^. In contrast, two of the grain-type soybeans displayed lower glucose concentrations of 0.6 μg·mL^-1^ and 1.5 μg·mL^-1^, while glucose was not detected in the two food-grade soybeans tested. Among the four sugars detected, fructose displayed the highest concentration in the total seed leachates, constituting 48.2% of the total sugar contents, while sucrose had the lowest concentration at 9.5%. Among the nine genotypes, R17-2965 (426.4 μg·mL^-1^) and V16-0523HP (416.7 μg·mL^-1^) released the highest amounts of sugars, while two food-grade soybeans, MFS 561 and MFS 48P1, had the lowest sugar leakage, measuring 2.6 μg·mL^-1^ and 3.1 μg·mL^-1^, respectively.

#### 3.4.2 Soluble amino acids

Fourteen amino acids were present in the leachate of the nine genotypes tested (**Figure 2B**). Tryptophan was not detectable in any genotype, while cysteine and valine were absent only in the leachate of V16-0688HP. Notably, serine was the most common amino acid accounting for 21.7% the total amino acid content in the leachates followed by glutamic acid at 14.5%. Conversely, histidine (1.8%) and phenylalanine (1.9%) were the lowest amino acids in the leachate.

Among the nine genotypes, two edamame genotypes, R17-2965 (110.7 mg·mL^-1^), followed by V16-0523HP (69.6 mg·mL^-1^), released the most amino acids in their leachate. These two genotypes were characterized by high serine and glutamic acid, concentrations of 22.8 mg·mL^-1^ and 15.3 mg·mL^-1^ for R17-2965 and 15.1 mg·mL^-1^ and 12.9 mg·mL^-1^ for V16-0523, respectively. In contrast, the grain-type soybean USG 5618v exhibited the lowest total amino acid leakage at 9.7 mg·mL^-1^, followed by the food-grade soybeans, MFS 48P1, which measured 10.03 mg·mL^-1^. Serine (2.3 mg·mL^-1^) and methionine (1.1 mg·mL^-1^) were the predominant amino acids in USG 5618v, while lysine (2.9 mg·mL^-1^) and serine (2.4 mg·mL^-1^) were the predominant amino acids in MFS 48P1.

#### 3.4.3 Mineral elements

Five mineral ions were detected in the soybean leachate, i.e., Ca, P, Na, K, Mg. Except Ca, P, all the chemical elements were present in all the genotypes tested (**Figure 2C**). Ca was notably absent in the leachate samples of the Ellis genotype, while P was absent in one of the edamame genotypes, i.e., V16-0688HP as well as all the food-grade or grain-type soybean genotypes tested. K displayed the highest concentration, constituting 91.6% of the total mineral content of all the leachate samples, followed by P, which accounted for 3.1% of the total mineral content, even though it was only present in several genotype leachates. In contrast, Na had the lowest share, making up only 0.7% of the total mineral contents.

Among the nine genotypes, R17-2965 (145.4 μg·mL^-1^) and V16-0523HP (139.9 μg·mL^-1^) released the highest mineral contents. K was the predominant mineral element in both genotypes, with concentrations of 128.7 μg·mL^-1^ for R17-2965 and 124.3 μg·mL^-1^ in V16-0523HP. Food-grade soybean MFS 48P1 and grain-type soybean Ellis had the lowest mineral leakage, measuring 20.2 μg·mL^-1^ and 24.7 μg·mL^-1^, respectively, with K being the predominant mineral in both as well, at 19.4 μg·mL^-1^and 24.1 μg·mL^-1^, respectively. Edamame genotypes, in general, exhibited higher mineral contents of Ca (1.0 μg·mL^-1^-3.4 μg·mL^-1^), K (55.3 μg·mL^-1^-128.7 μg·mL^-1^), and Mg (1.6-5.1 μg·mL^-1^) compared to other types of soybeans, which had Ca (<0.6 μg·mL^-1^), K (19.4 μg·mL-1-31.7 μg·mL^-1^), and Mg (<0.8 μg·mL^-1^). In contrast, all genotypes contained Na, with values varying by genotype, ranging from 0.3 μg·mL^-1^ to 0.6 μg·mL^-1^, but no difference was observed between soybean types. Except V16-0688HP, all edamame genotypes contained P, with concentrations ranging from 0.2 μg·mL^-1^to 8.3 μg·mL^-1^, but P was not detected in either food-grade or grain-type soybeans.

### 3.5 The correlation between seed physiological traits and seed performance

The total seed sugar content displayed a slightly positive correlation with MTG (r=0.5, P<0.01), as well as with EC (r=0.5, P<0.01) and seed size (r=0.5, P<0.01) (**Supplementary Table S3**). Among the three major sugars of soybean seeds (i.e., sucrose, raffinose, and stachyose), sucrose exhibited a stronger correlation with MTG (r=0.6, P<0.01) than raffinose, while no significant correlation observed between seed performance and seed stachyose content nor the ratio of raffinose to sucrose. Additionally, EC values showed a significant correlation with MTG (r=0.7, P<0.01), although no significant correlations were observed when the EC value was divided by seed weight. Furthermore, seed size showed significant positive correlations with MTG (r=0.9, P<0.01) in a laboratory. However, no correlation was found between seed size and field emergence in this study.

There was high correlation coefficient between EC and total sugars (r=0.9; P<0.01), total amino acids(r=0.9), and total minerals (r=1) in the leachates (**Supplementary Table S4**). When examining individual soluble sugars in the leachate, all sugars, except for raffinose, showed significant positive correlations with EC (r>0.8; P<0.01). For amino acids, all 17 amino acids contributed to EC with correlations greater than 0.8, except for tryptophan, arginine, cysteine and lysine. All mineral elements, except for sodium, contributed to conductivity with correlations exceeding 0.90. Potassium exhibited the highest correlation coefficient at r=1, signifying a high association with EC, whereas sodium did not exhibit a relationship with EC.

### 3.6 Emergence responses across temperatures

#### 3.6.1 Optimal temperatures

**Figure 3:**
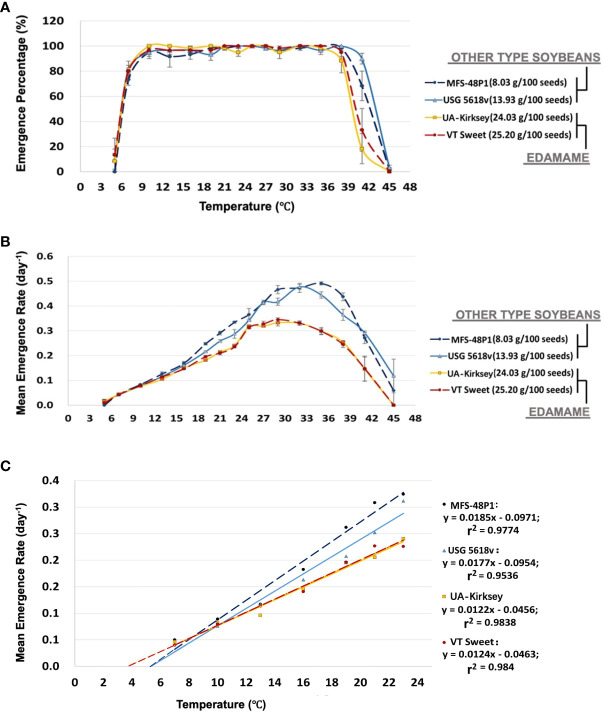
Emergence percentage of two commercial edamame and two other types of soybean cultivars at different temperatures **(A)**. Mean maximum temperatures were estimated graphically as those that reduced emergence to 50% and were summarized in **Table 2**. Values shown are means ± standard error from four replications of 15 seeds per replication. The tested temperatures included 5°C, 7°C, 10°C, 13°C, 16°C, 19°C, 21°C, 23°C, 25°C, 27°C, 29°C, 32°C, 35°C, 38°C, 41°C, and 45°C. MFS 48P1 belongs to food-grade soybean and USG 5618v belongs to grain-type soybean. Mean emergence rate of four cultivars across temperatures **(B)**. Optimal temperature(s) (T_o_) for emergence are summarized in **Table 2** and determined as the temperatures where the highest emergence percentage and emergence speed occurred simultaneously. Values shown are means ± standard error from four replications of 15 seeds per replication. Representative linear regression equations from one replication, calculated over the range of temperatures for four cultivars and used in the calculation of mean minimum temperatures (T_b_) and thermal time to emergence (θ_T_) in **Table 2** **(C)**. T_b_ values were determined by extrapolating plots of mean emergence rate versus temperature (T) to the intercept on the abscissa. θ_T_ values were the reciprocal of the slopes of the regression.

To assess variations in emergence responses to temperature, we analyzed the emergence percentage and speed of two representative edamame cultivars, along with one each from grain-type and food-grade soybeans, across seventeen temperatures from 5°C to 45°C. The seedling emergence percentage of all types of soybeans exhibited a sharp increase as the temperature rose from 7 to 10°C (**Figure 3A**). From13 to 35°C, the emergence exceeded 90% for all cultivars. However, percentages sharply declined above 38°C. Notably, edamame declined significantly more than other types of soybeans. Edamame exhibited emergence of less than 50% at 41°C, whereas both the grain-type and food-grade soybean cultivars emerged above 60% at this temperature. No emergence occurred for any soybean types above 45°C (**Figure 3A**).

The emergence rate (ER) increased linearly over a temperature range from 7°C to 23°C for all the cultivars (**Figure 3B**). The food-grade and grain-type soybean cultivars displayed a consistent linear increase until reaching 32°C, while the two edamame cultivars rapidly increased in ER from 23°C to 25°C, followed by a plateau between 25°C and 32°C. Subsequently, there was a gradual decline from 32°C to 37°C. Both edamame and the other types of soybeans exhibited a sharp decrease in ER at even higher temperatures (**Figure 3B**). For edamame, the highest emergence rate of 0.34 seeds/day occurred at 29°C. Conversely, the food-grade and grain-type soybeans achieved their peak emergence rates of nearly 0.5 seeds/day at 35°C and 32°C, respectively.

When considering both the emergence percentage and rate over a wide temperature range, the optimal temperature range for edamame seedling emergence fell between 25-33°C. Within this range, edamame seeds could emerge in approximately 3 days with an emergence percentage exceeding 90%. In contrast, for both food-grade and grain-type soybeans, the optimal temperature range was 27-36°C when both types achieved >90% emergence with an emergence rate exceeding 0.4 seeds/day (**Figures 3A B**; **Table 2**).

**Table 2:** Base (T_b_), ceiling (T_m_) and optimal (T_o_) temperatures, and thermal time (θ_T_), for seedling emergence of edamame and other type soybean cultivars.

Cultivar	T_b_ (°C)	T_o_ (°C)	T_m_ (°C)	ϴ_T_ (°C d)
*Edamame*				
UA Kirksey	3.9b*	25-33	39.4b	81.2a
VT Sweet	3.5b	25-33	40.7ab	82.1a
*Food Grade Soybean*				
MFS 48P1	5.5a	27-36	41.8ab	54.4b
*Grain Type Soybean*				
USG 5618v	4.9a	27-38	42.4a	64.4b
*Total Mean*	4.5	26-35	41.08	70.5

*Values shown are means from four replications of 15 seeds per replication. Means followed by the same letters are not significantly different according to Tukey’s HSD (P <0.05) within the data collected in the same column.

#### 3.6.2 Effects of temperature on germination base and maximum temperatures as well as thermal times

T_b_ values for each soybean cultivar were calculated through linear regression of germinate rate versus temperature over a range of 7–23°C (**Figure 3C**). Interestingly, the T_b_ values for the edamame cultivars (approximately 4°C) consistently remained lower compared to those observed for other soybean types (approximately 5-6°C) (**Table 2**). Additionally, there were significant differences in thermal time between edamame and the other types. Thermal times were higher for edamame (approximately 80°C d) in comparison to grain-type and food-grade cultivars (i.e., 64.4°C d and 54.4°C d, respectively). Notably, no significant differences were observed within the edamame group or among the two other soybean types (**Table 2**).

Determination of T_m_ was complicated by the abrupt decline in emergence percentages above 40°C for all types. T_m_ was estimated by comparing the temperatures that inhibited emergence by 50% (**Figure 3A**). ANOVA revealed differences in T_m_ among cultivars, instead of soybean types, and T_m_ values ranged from 42.4°C to 39.4°C (**Table 2**).

## 4 Discussion

All 13 edamame genotypes germinated to percentages exceeding 80% in the AOSA tests and exceeded 90% in LabField™ seedling emergence tests at a constant 25°C in a laboratory (**Table 1**). Edamame genotypes displayed rapid seedling emergence within 3-4 days in soil of the LabField™ emergence test, in contrast to their mean germination time of 4 to 6 days in laboratory germination tests due to improved seed to soil contact and warm soil temperatures. Continuous soil contact increases water absorption and seed temperature transfer compared to germination testing using paper substrates in many standardized laboratory germination tests. The difference between soil and paper substrate germination was less obvious for food-grade and grain-type soybeans, possibly due to their smaller seed size. In laboratory tests, there were no significant differences in the final germination and emergence percentages among edamame genotypes, nor between edamame and other soybean types, indicating that there were no genetic or physiological limitations to edamame seed quality that reduced viability of vigor compared to other types of soybeans.

The final stands of edamame and the other soy genotypes exceeded 70% in the field tests in 2021. Many edamame genotypes, including existing cultivars UA Kirksey and VT Sweet, surpassed 80% final stands. Notably, there were no discernible differences in field performance between edamame and other soybean types, whether in terms of the final percentage or emergence rate. This contrasts with previous studies that reported poor field emergence in edamame despite high laboratory germinability (Williams, 2015). Our research confirmed that edamame genotypes have the potential for high stand establishment under favorable field conditions when initial seed quality is good.

Edamame seeds are considerably larger, ranging from 65% to 100% larger than grain-type soybean seeds (Crawford and Williams, 2018). Consequently, they require more water and take longer to complete imbibition (**Supplementary Figure S3**). Our findings indicate that seed size has a significant positively correlation with germination time (r=0.9; P < 0.01), but it does not significantly reduce final emergence percentages. These results are consistent with the findings of Crawford and Williams (2019). Interestingly, the results of the LabField ™ seedling test and the field study of this work also reveals that the negative impact of larger seed size on edamame germination can be alleviated when seeds are placed in warm soil with sufficient water. This may be attributed to the larger surface area of bigger seeds, which enhances water absorption rates and shortens germination time when the seeds are fully enveloped in warm, moist soil.

For many decades, the impact of seed size on soybean seedling emergence has been a subject of investigation, resulting in a substantial body of literature with conflicting findings. Some authors have suggested that larger seeds had lower germination and emergence especially when subjected to challenging field conditions like low temperatures, anoxia, crusted soils, or compacted soils, due to greater physical soil resistance larger seeds encounter as their larger cotyledons emerge (Edwards and Hartwig, 1971; Hoy and Gamble, 1985; Liu et al., 2012; Adebisi et al., 2013; Kering and Zhang, 2015). However, some studies have found positive effects of larger seed size on emergence as they provide more reserves to ensure sustained growth until true leaves develop (Burris et al., 1973; Morrison and Xue, 2007; Madanzi et al., 2010; Rezapour et al., 2013). Finch-Savage and Bassel (2016) also noted that large seeds have larger cells, which can generate more force and perform better under stress conditions, explaining their advantage. Other researchers have failed to detect any clear relationship between soybean seed size and germination or field emergence (Fontes and Ohlrogge, 1972; Johnson and Luedders, 1974; Smith and Camper, 1975). In this study, we also found that seed size did not impact field emergence, either in terms of the final percentage nor the speed of emergence when the conditions are suitable. Consequently, it is likely that seed quality and the conditions of the seedbed during crop growth exert a more dominant influence on edamame emergence than variations in seed size.

The passive release of electrolytes begins with initial seed imbibition and continues until germination begins (Lamichhane et al., 2018). Electrolyte leakage is a popular assessment of legume seed quality because high electrical conductivity is often correlated with membrane damage and leached electrolytes (Schoettle and Leopold, 1984). As seeds age, they release more electrolytes during imbibition, leading to reduced seed vigor due to the loss of low molecular weight metabolites from cotyledonary cells. A study by Hoy and Gamble (1985) found that larger seeds leaked more electrolytes, which correlated with lower seed vigor. In this research, we confirmed that edamame seeds released electrolytes more rapidly and exhibited higher total conductivity compared to food-grade and grain-type soybean seeds, which are smaller. However, when conductivity values were normalized by seed weight, no significant differences were observed between edamame and other soybean types, and no correlations were found between adjusted EC and seed germination.

Electrolytes typically comprise charge-carrying seed metabolites, such as amino acids, flavonoids, sterols, and salts that exist in solution as ions (Schiltz et al., 2015). Edamame electrolyte leakage curves were bimodal with rapid leakage shortly after the onset of imbibition followed by a lag phase and then more leakage. This suggests that initial leakage was due to a combination apoplastic and membrane leakage resulting from initial hydration (**Supplementary Figure S2**, Bradford, 1994). Apoplastic solutes accumulate during seed fill and residual amounts vary with environmental conditions and seed genotype. After a plateau phase solutes leakage again increased possibly due to anoxia of submerged seeds and failure of membrane damage to be fully repaired (**Supplementary Figure S2**). Additionally, electrolyte and solute leakage attracts and feeds degradative soilborne pathogens such as *Pythium ultimum* and *Rhizoctonia* spp. responsible for soybean seed rot and seedling damping-off (Matthews and Powell, 1968; Parera et al., 1996; Kageyama and Nelson, 2003; Finch-Savage and Bassel, 2016). Greater total leakage of electrolytic solutes from large-seeded edamame cultivars likely contribute to soil-borne diseases. Treating edamame seeds with fungicide or biologicals like *Trichoderma harzianum* may provide the extra protection needed to safeguard vulnerable emergent seedlings from soilborne pathogenic attack brought on by seed leakage especially in poorly drained disease-prone soils (Williams & Bradley, 2017; Pimentel et al., 2022).

Previous studies have reported that grain type soybean germination rates can vary significantly, ranging from 2 weeks or more in cold soil (10°C or less) to about 4 days under optimum soil temperatures (27–30°C) (Kumudini, 2010). Additionally, Sánchez et al. (2005) conducted a study comparing the seedling emergence of edamame under various 4-day/night temperature regimes (15.6/10, 21.1/15.6, 26.7/21.1, and 32.2/26.7°C) on 12-hour cycles. Sánchez et al. (2005) concluded that 21.1/15.6°C was the optimal temperature for edamame emergence, which is notably lower than the optimal temperature suggested for grain type soybean. Similarly, in this work, edamame exhibited a lower optimal emergence temperature range (25-33°C) in which emergence percentages could surpass 90%, in contrast to grain soybeans (27-38°C) and food-grade soybean cultivars (27-36°C). At the optimal temperature range, edamame typically emerges in approximately 3-3.5 days after sowing, while grain-type and food-grade cultivars typically emerge slightly faster, within 2-2.5 days, assuming no other limiting factors for emergence. This difference suggests the potential for edamame to be planted earlier than the other two soybean types, to ensure a more reliable stand establishment.

Our results generally agree with prior research, which reported the base, optimum, and maximum temperatures for grain soybean as 4, 30, and 40°C, respectively (Hatfield and Egli, 1974). In this work, neither edamame nor other soybean types emerged at temperatures below 4°C. However, edamame seeds displayed a lower base temperature compared to other soybean types (**Table 2**), possibly due to their greater energy reserves and a larger volume-to-surface area ratio, which reduces heat loss (Fontes and Ohlrogge, 1972). The maximum temperature for all the soybean types tested was around 40°C. Our results also confirmed that edamame deteriorate faster at the high temperature, compared with other type soybean, which was supported by the previous study which reported that larger soybean seeds deteriorate faster than smaller ones (Shelar et al., 2008). Furthermore, edamame demonstrated higher thermal times required for emergence compared to other types of soybeans, indicating that edamame may require a longer duration of accumulated heat for germination. This could be attributed to edamame aging more rapidly.

Sucrose was found in greatest quantity in soybean seeds (which type grain, edamame or food grade) followed by stachyose and raffinose representing 4.9%, 3% and 1.5% of mature seeds (Escamilla et al., 2019). In the current study, edamame, contained 5.1% sucrose in mature seeds compared to only 0.7 in stachyose. This is consistent with the fact that sucrose contributes to the sweetness of edamame, whereas raffinose and stachyose are undesirable due to their indigestibility and potential to cause flatulence or diarrhea (Escamilla et al., 2019). The sugar content of seeds, primarily sucrose, was positively correlated with longer MTG (r=0.6, P<0.01, **Supplementary Table S3**); however, this effect existed primarily in standardized laboratory tests on paper and there was no correlation in laboratory or field soil emergence tests under favorable conditions. Conversely, the concentrations of raffinose and stachyose in mature seeds were unrelated to soybean seed germination (**Supplementary Table S3**, Dierking and Bilyeu, 2009). High sucrose content is a desirable trait for edamame, but seeds with higher sucrose content may experience longer germination times under stressful conditions, posing a risk of seed decay due to biotic and abiotic stressors in soil. Moreover, seeds with higher sucrose content tend to leak more sucrose during germination (**Supplementary Table S4**), as observed in this study, promoting the growth of seedborne pathogens, and increasing the potential for disease occurrence. Therefore, it is advisable to sow edamame when soil conditions are optimal.

In this study, we employed LabField™ tests for seed quality evaluation, which proved to be a more accurate assessment in a laboratory setting compared to traditional germination tests using paper towels. Additionally, LabField™ was utilized to effectively evaluate the influence of temperature on edamame seed emergence and to identify the optimal temperature for seedling growth. The controlled environment of the LabField™ tests allows for the specific assessment of individual factors, such as temperature, on germination performance, thereby isolating these from the complex edaphic influences typically encountered in field trials. Moreover, to achieve a comprehensive evaluation of crop emergence across diverse environments, extensive multi-year field trials are essential. These trials are crucial for understanding how temperature interacts with other environmental factors, such as soil moisture and crusting, which significantly impact seed establishment.

## 5 Conclusions

In conclusion, the burgeoning interest in edamame represents a promising opportunity for growers focused on vegetable crop production. This study provides a foundational understanding of the germination and emergence processes of edamame, examining edamame-specific physiological traits and their potential interaction with crucial environmental factors that influence emergence. Contrary to common beliefs that attribute poor edamame stands to genetic factors, our findings reveal that the more typical causes include poor seed quality and adverse field conditions rather than inherent issues with edamame seed quality itself. Furthermore, our research highlights that the larger size of edamame seeds contribute to slower emergence due to the extension time required for hydration and emergence. Therefore, it is advisable to sow edamame under optimal soil and environmental conditions to encourage rapid emergence and to improve seedling establishment.

## Data availability statement

The original contributions presented in the study are included in the article/**Supplementary Material**. Further inquiries can be directed to the corresponding authors.

## Author contributions

XL: Data curation, Formal analysis, Investigation, Writing – original draft. KL: Data curation, Investigation, Writing – review & editing. SR: Funding acquisition, Supervision, Writing – review & editing. LR: Data curation, Methodology, Writing – review & editing. BZ: Funding acquisition, Project administration, Resources, Supervision, Writing – review & editing. GW: Conceptualization, Funding acquisition, Investigation, Methodology, Resources, Supervision, Writing – review & editing.

## Supplementary material

Supplementary Figure 1 Minimum, average, and maximum soil temperatures recorded each day during the 24 days following the planting of edamame in the field.

Supplementary Figure 2 Individual electrical conductivity time course of six representative edamame and other types of soybeans over the 36-hour soaking period. Mean values and standard error deviations for each genotype were recorded at three-hour intervals during the course.

Supplementary Figure 3 Difference between two edamame cultivars and two other type soybeans during seed hydration at 25°C. Values showed are means ± standard error from four replications of 25 seeds per replication.
