# Supplementary figures and images for: Seed physiological traits and environmental factors influence seedling establishment of vegetable soybean (Glycine max L.)
Source: Front Plant Sci. 2024 Jun 3;15:1344895. doi: 10.3389/fpls.2024.1344895 (PMC11180749; doi:10.3389/fpls.2024.1344895)

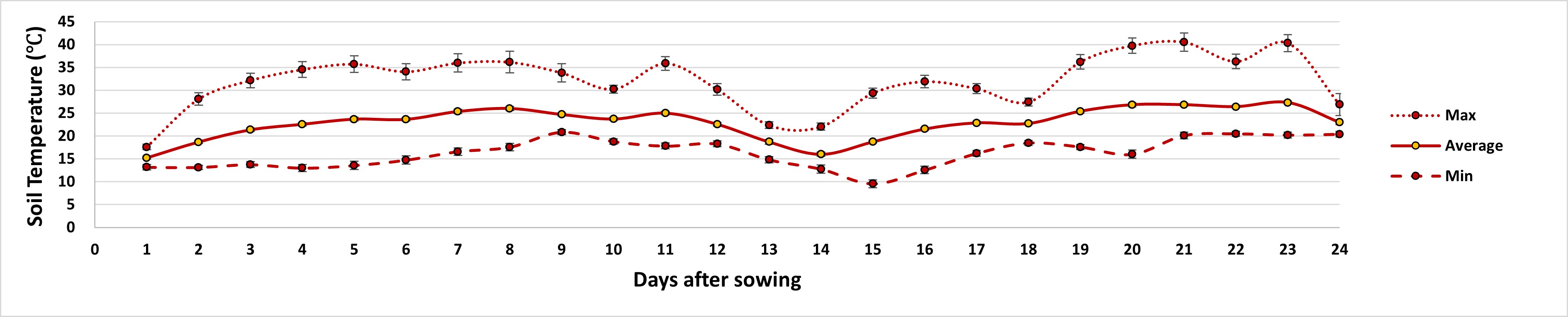

Supplement: Supplementary Figure 1 — Minimum, average, and maximum soil temperatures recorded each day during the 24 days following the planting of edamame in the field. [file Image_1.jpeg]

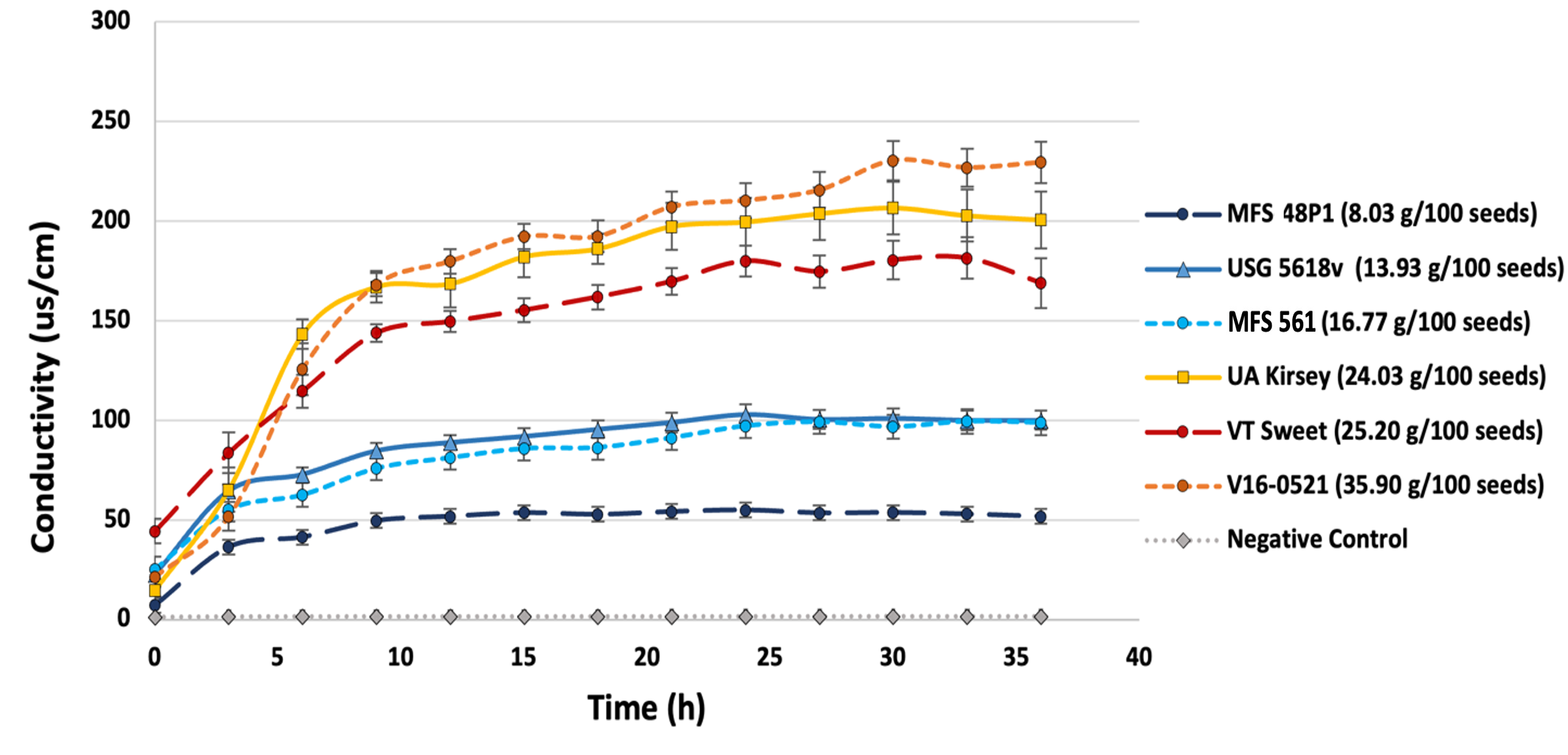

Supplement: Supplementary Figure 2 — Individual electrical conductivity time course of six representative edamame and other types of soybeans over the 36-hour soaking period. Mean values and standard error deviations for each genotype were recorded at three-hour intervals during the course. [file Image_2.png]

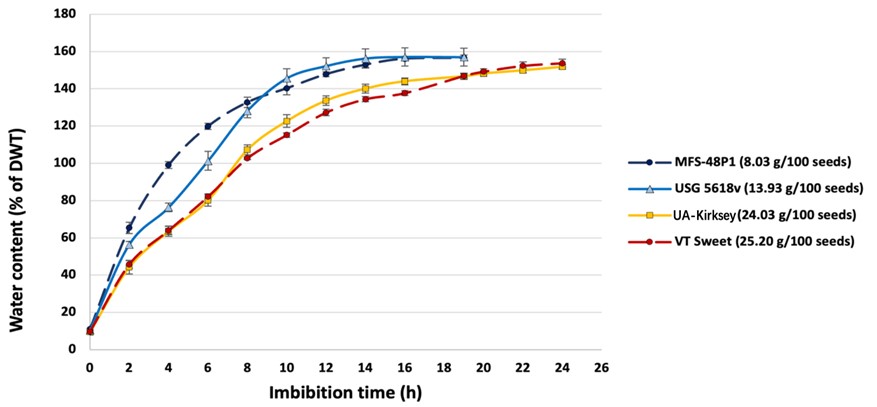

Supplement: Supplementary Figure 3 — Difference between two edamame cultivars and two other type soybeans during seed hydration at 25°C. Values showed are means ± standard error from four replications of 25 seeds per replication. [file Image_3.jpeg]
